# 548 Diabetic Foot Burns and Amputation Rates: A Systematic Review and Meta-analysis

**DOI:** 10.1093/jbcr/irae036.182

**Published:** 2024-04-17

**Authors:** Brigette Cannata, Nicolas Malkoff, Deborah Choe, Justin Gillenwater

**Affiliations:** Keck School of Medicine of USC, Staten Island, NY; Keck School of Medicine of USC, Carlsbad, CA; Keck School of Medicine of USC, Los Angeles, CA; Keck Medicine of USC, Los Angeles, CA; Keck School of Medicine of USC, Staten Island, NY; Keck School of Medicine of USC, Carlsbad, CA; Keck School of Medicine of USC, Los Angeles, CA; Keck Medicine of USC, Los Angeles, CA; Keck School of Medicine of USC, Staten Island, NY; Keck School of Medicine of USC, Carlsbad, CA; Keck School of Medicine of USC, Los Angeles, CA; Keck Medicine of USC, Los Angeles, CA; Keck School of Medicine of USC, Staten Island, NY; Keck School of Medicine of USC, Carlsbad, CA; Keck School of Medicine of USC, Los Angeles, CA; Keck Medicine of USC, Los Angeles, CA

## Abstract

**Introduction:**

Diabetes mellitus (DM) is one of the most common chronic metabolic diseases. Comorbid peripheral neuropathy (PN) and peripheral vascular disease (PVD) puts this population at risk for foot burns and poor wound healing outcomes. There is a lack of consensus in the literature regarding the optimal approach for management of these unique injuries. The aim of this study is to systematically identify studies reporting on diabetic foot burns and critically evaluate outcomes including amputation rates among patients managed operatively versus non operatively.

**Methods:**

A comprehensive search of PubMed, Embase, and Web of Science was completed. Screening was performed by two independent reviewers. Primary research studies published between 1980 to 2023 that discussed outcomes of foot burns in adult patients with DM were included. Studies that included pediatric (< 18 years old) or animal subjects, had inaccessible full texts, or were written in a language other than English were excluded. All conflicts were resolved collaboratively. The included studies were critically appraised using Newcastle-Ottawa Scale (NOS), JBI critical appraisal tool for analytical cross-sectional studies, or a published methodological tool. Results are presented using descriptive statistics of aggregated data.

**Results:**

The database search yielded 2,402 non-duplicate papers, which was narrowed to 105 full texts following initial screening. A total of 35 papers met inclusion criteria, 5 of which were removed for poor scores on critical appraisal. Nine papers were included for meta-analysis, including seven retrospective comparative analyses, one cross-sectional study, and one retrospective chart review. In sum, there were 1798 diabetic foot burn patients included for analysis. Mean age was 58.2 years (SD 4.12) and 73.1% (n = 1,314) were male. A total of 15.7% (n = 283) of patients were surgically managed, which included debridement (3.7%, n = 66), grafting (8.2%, n = 147), flap (0.2%, n = 3), and primary amputation (7.1%, n = 127). Secondary amputation rate, defined as amputation following initial surgery, was 4.9%, (n = 14). The overall amputation rate of the cohort was 7.8% (n = 141). Other complications included infection (4.0%, n = 72), osteomyelitis (1.9%, n = 34), and graft failure (8.2%, n = 12). Only 1 study reported functional status at last visit.

**Conclusions:**

Diabetic foot burn patients that are managed operatively experience increased morbidity including graft failure, infection, and need for additional amputation. This can have damaging effects on functional outcomes after injury.

**Applicability of Research to Practice:**

The global diabetic population is growing and vulnerable to poor health outcomes, driving the need for research on diabetic foot burns to help providers reach consensus on best management practices.

